# The thermal sensation threshold and its reliability induced by the exposure to 28 GHz millimeter-wave

**DOI:** 10.3389/fnins.2024.1331416

**Published:** 2024-02-27

**Authors:** Akiko Yuasa, Shintaro Uehara, Kazuki Ushizawa, Sachiko Kodera, Norika Arai, Akimasa Hirata, Yohei Otaka

**Affiliations:** ^1^Department of Rehabilitation Medicine I, School of Medicine, Fujita Health University, Toyoake, Aichi, Japan; ^2^Faculty of Rehabilitation, School of Health Sciences, Fujita Health University, Toyoake, Aichi, Japan; ^3^Department of Electrical and Mechanical Engineering, Nagoya Institute of Technology, Nagoya, Aichi, Japan; ^4^Center of Biomedical Physics and Information Technology, Nagoya Institute of Technology, Nagoya, Aichi, Japan

**Keywords:** thermal sensation, millimeter wave, perception threshold, skin temperature, reliability

## Abstract

The application of 28 GHz millimeter-wave is prevalent owing to the global spread of fifth-generation wireless communication systems. Its thermal effect is a dominant factor which potentially causes pain and tissue damage to the body parts exposed to the millimeter waves. However, the threshold of this thermal sensation, that is, the degree of change in skin temperature from the baseline at which the first subjective response to the thermal effects of the millimeter waves occurs, remains unclear. Here, we investigated the thermal sensation threshold and assessed its reliability when exposed to millimeter waves. Twenty healthy adults were exposed to 28 GHz millimeter-wave on their left middle fingertip at five levels of antenna input power: 0.2, 1.1, 1.6, 2.1, and 3.4 W (incident power density: 27–399 mW/cm^2^). This measurement session was repeated twice on the same day to evaluate the threshold reliability. The intraclass correlation coefficient (ICC) and Bland–Altman analysis were used as proxies for the relative and absolute reliability, respectively. The number of participants who perceived a sensation during the two sessions at each exposure level was also counted as the perception rate. Mean thermal sensation thresholds were within 0.9°C–1.0°C for the 126–399 mW/cm^2^ conditions, while that was 0.2°C for the 27 mW/cm^2^ condition. The ICCs for the threshold at 27 and 126 mW/cm^2^ were interpreted as poor and fair, respectively, while those at higher exposure levels were moderate to substantial. Apart from a proportional bias in the 191 mW/cm^2^ condition, there was no fixed bias. All participants perceived a thermal sensation at 399 mW/cm^2^ in both sessions, and the perception rate gradually decreased with lower exposure levels. Importantly, two-thirds of the participants answered that they felt a thermal sensation in both or one of the sessions at 27 mW/cm^2^, despite the low-temperature increase. These results suggest that the thermal sensation threshold is around 1.0°C, consistent across exposure levels, while its reliability increases with higher exposure levels. Furthermore, the perception of thermal sensation may be inherently ambiguous owing to the nature of human perception.

## 1 Introduction

Electromagnetic fields have played a pivotal role in humanity’s progress, from daily living to the global industrial infrastructure. Particularly, the spread of fifth-generation wireless communication systems (5G) has been deployed from 2020. In 5G, different frequency bands are used as compared to conventional wireless communications system. Specifically, in addition to 3.5 GHz, a higher frequency band of 24–28 GHz is used (European Commission, 2018), which is different in different countries (Matinmikko-Blue et al., 2020) (24–28 GHz band, hereafter millimeter is used to refer to 5G millimeter). A key adverse effect of millimeter waves is its thermal effect, as its exposure leads to localized heating, causing thermal tissue damage and pain [The Institute of Electrical and Electronics Engineers (IEEE), 2019b; International Commission on Non-Ionizing Radiation Protection (ICNIRP), 2020]. However, the threshold of the thermal sensation of millimeter waves has not been assessed to date, which is the temperature at which the first subjective response to the thermal effects of the millimeter waves occurs. A large number of studies have investigated the thermal or pain thresholds in animals or human subjects in ocular tissues (reviewed by Foster et al., 2021), yet few have investigated these thresholds in other body parts. In particular, to the best of our knowledge, only two studies have examined the effects on the skin, and they were specifically limited to the back of the human body (Blick et al., 1997; Walters et al., 2000). The permissible exposure limits have been prescribed in the international guidelines published by the Institute of Electrical and Electronics Engineers (IEEE) C95.1 standard [The Institute of Electrical and Electronics Engineers (IEEE), 2019a] and the International Commission on Non-Ionizing Radiation Protection (ICNIRP) (2020), which are mentioned by the World Health Organization (WHO). A recent review specifies that the exposure restrictions for millimeter wave are largely based on electromagnetic and thermal modeling studies (Hirata et al., 2021) due to the lack of data from radiofrequency exposure above 6 GHz (Foster et al., 2021). Given the prevalence of millimeter waves in our surroundings, there is an urgent need to investigate the effects of millimeter waves on the skin.

To the best of our knowledge, no previous study has investigated the reliability of measuring the thermal sensation threshold evoked by electromagnetic energy. The reliability of these measurements should therefore be ascertained when investigating thermal sensation thresholds in humans. The perception of sensation is highly subjective for its evaluation in individuals; the psychological and physical state of an individual easily influences the evaluation (Siao and Cros, 2003). Furthermore, the sensation of warmth evoked by the exposure to millimeter waves is relatively less clear than other sensations such as tingling or pain. In fact, with thermal quantitative sensory testing, the reliability of warmth detection is shown to be lower than that of heat pain (Moloney et al., 2011). To date, several methods have been used to measure the thermal sensation threshold induced by the electromagnetic fields, which are step series of stimuli (Walters et al., 2000), method of limits (Chatterjee et al., 1986), or method of constant stimuli (Nakatani-Enomoto et al., 2019; Uehara et al., 2023). However, these methods are known to have some disadvantages, such as variability of the results (i.e., weak reliability) due to increasing or changing exposure levels during a trial, and time consumption that affects participant’s attention (Shy et al., 2003). Considering these points, our method applied a constant level of exposure to the target body part with a time limit.

To preliminarily investigate the adverse health effects of millimeter waves on the human body, we aimed to measure the threshold of the thermal sensation evoked by the exposure to millimeter waves and examined the reliability of the measurement. Since a computational model showed the varying effect of the electrical power on the degree of body temperature changes (Sasaki et al., 2017), we hypothesized that the reliability would vary between the levels of exposed electromagnetic power density. To test this hypothesis, we explored the thermal sensation threshold using five different levels of incident power density (IPD), which is the external physical quantity defied as the (exposure) reference levels [The Institute of Electrical and Electronics Engineers (IEEE), 2019a; International Commission on Non-Ionizing Radiation Protection (ICNIRP), 2020].

## 2 Materials and methods

### 2.1 Participants

Twenty healthy adults [mean age, 35.2 years; standard deviation (SD), 5.0; four females] participated in this study. All participants provided written informed consent for participation in accordance with the 1964 Declaration of Helsinki, as revised in 2013. The inclusion criteria were: no history of diabetes mellitus or brain, neuromuscular, and psychiatric diseases. The exclusion criteria were as follows: (1) a history of receiving medical treatments that have the potential to influence sensation and perception or currently receiving such treatments, (2) having a cardiac pacemaker, (3) being pregnant, and (4) having eczema or skin rashes on the hand. This study was approved by the Ethics Review Committee, Fujita Health University (approval no. HM20-430).

### 2.2 Equipment

**Figure 1:**
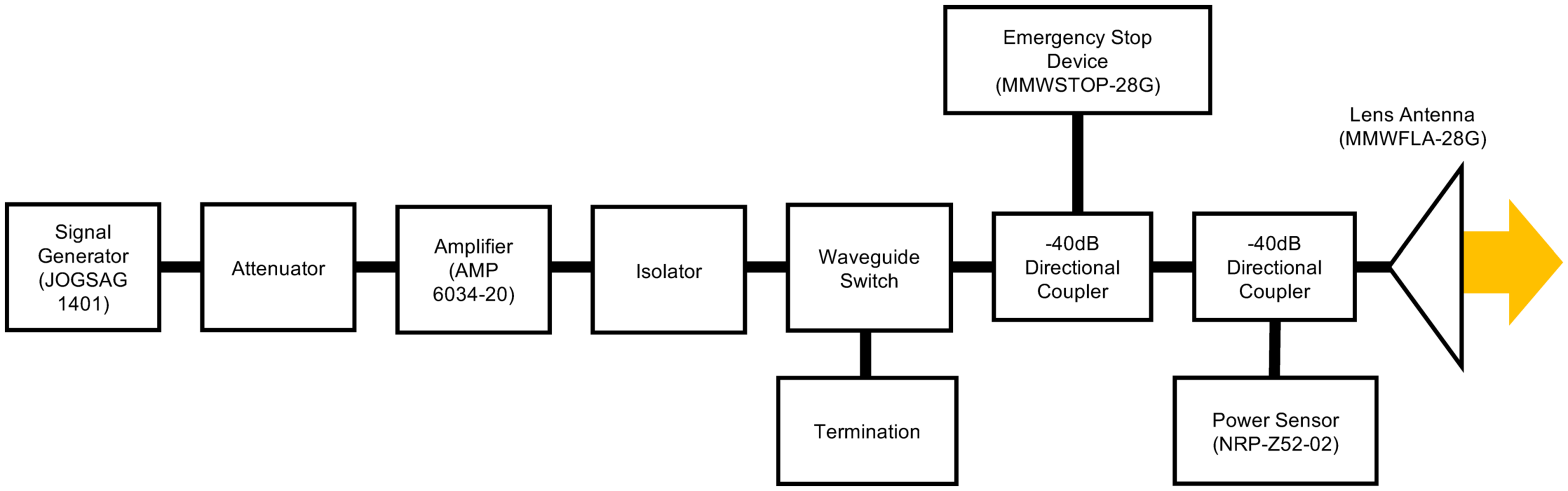
Block diagrams of the developed exposure equipment. The continuous and sinusoidal wave at 28 GHz via a lens antenna was irradiated to the fingertip.

**Figure 2:**
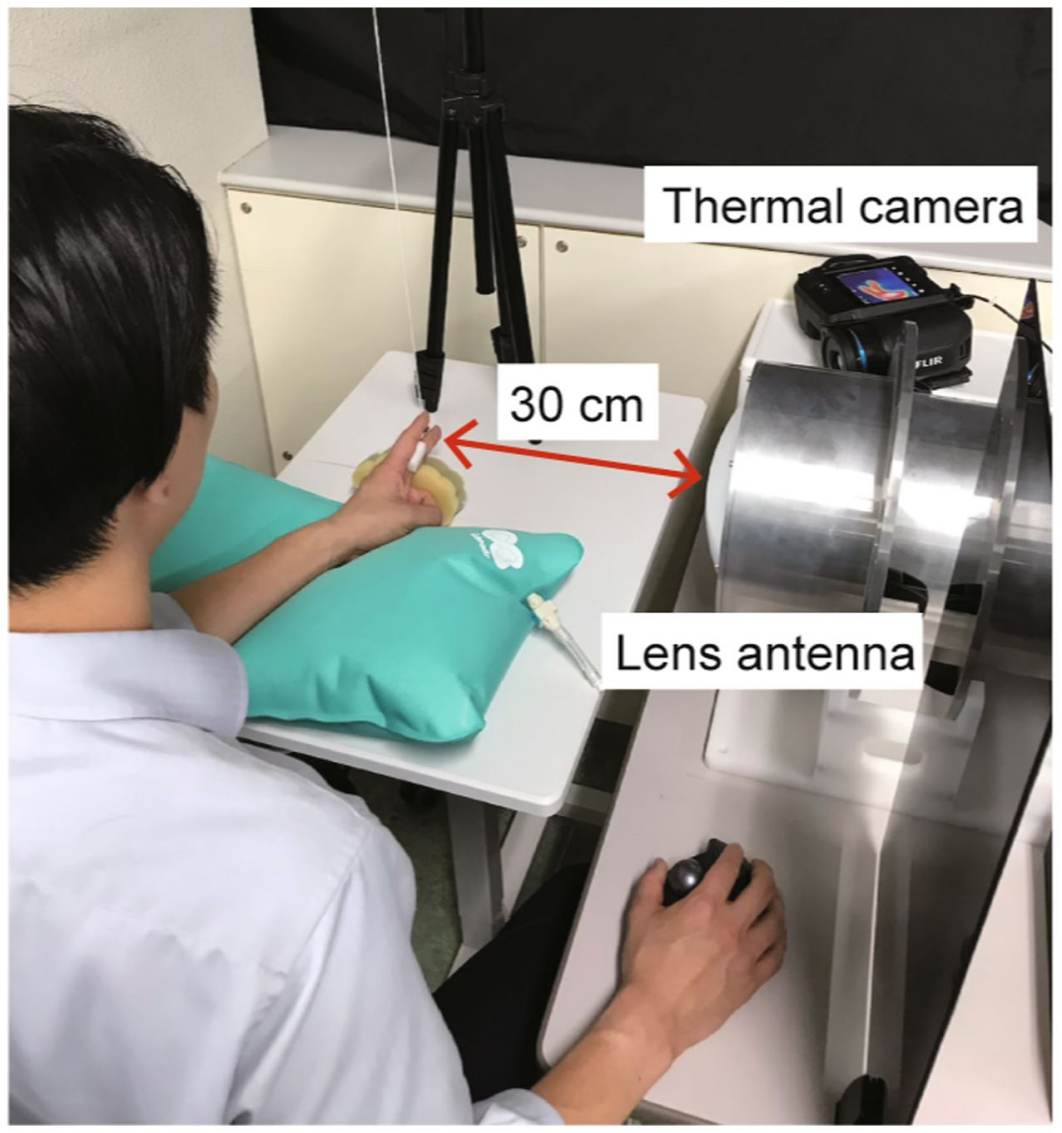
Experimental set-up. The fingertip of the left middle finger was set at 30 cm from the center of the lens antenna. The skin temperature of the fingertip hotspot was recorded using a thermal camera placed on the right side of a participant.

The continuous and sinusoidal millimeter waves were delivered to the participants at a frequency of 28 GHz on the fingertip of the left middle finger (as the targeted body part), and the thermal sensation threshold was measured. The equipment used to produce millimeter waves consisted of a signal generator (JOGSAG1401; SAF Tehnika, Riga, Latvia), power amplifier (AMP 6034-20; Exodus Advanced Communications, Las Vegas, NV, United States), lens antenna (MMWFLA-28G; Oshima Prototype Engineering, Japan), and the emergency stop device (MMWSTOP-28G; Oshima Prototype Engineering, Japan) (Figure 1). The power intensity into the antenna was adjusted by the attenuator and monitored by the power sensor (NRP-Z52-02; Rohde & Schwarz, Germany) during exposure. The isolator was embedded to avoid the reflected waves to the amplifier. Millimeter wave irradiation was turned on and off using a waveguide switch and a termination, minimizing fluctuations in the operating sound of the exposure equipment due to the presence or absence of irradiation. The emergency stop device embedded via a coupler operates the amplifier to stop the millimeter wave irradiation when a certain power intensity is exceeded, or if the participant feels unsafe. The distance between the center of the lens antenna and the target fingertip was set to 30 cm (Figure 2). The IPD at a distance of 30 cm from the antenna surface was measured using a field strength meter (SMP2 + WPF60, Wavecontrol S. L., Spain). With the center of the horn antenna as the origin, the IPD was measured at points along the horizontal axis ranging from −30 to +30 mm, with intervals of 5 mm. The measurements were repeated four times and were subsequently averaged for evaluation. The skin surface temperature was measured using a thermal camera (FLIR T530; Teledyne FLIR, Wilsonville, OR, United States) with a spatial resolution of 320 × 240 pixels at 30 frames/s. The skin temperature of the hotspot was recorded before, during, and after the exposure to millimeter waves. A thermal camera was placed on the right side of each participant (Figure 2).

### 2.3 Computational method of electromagnetic field

**Figure 3:**
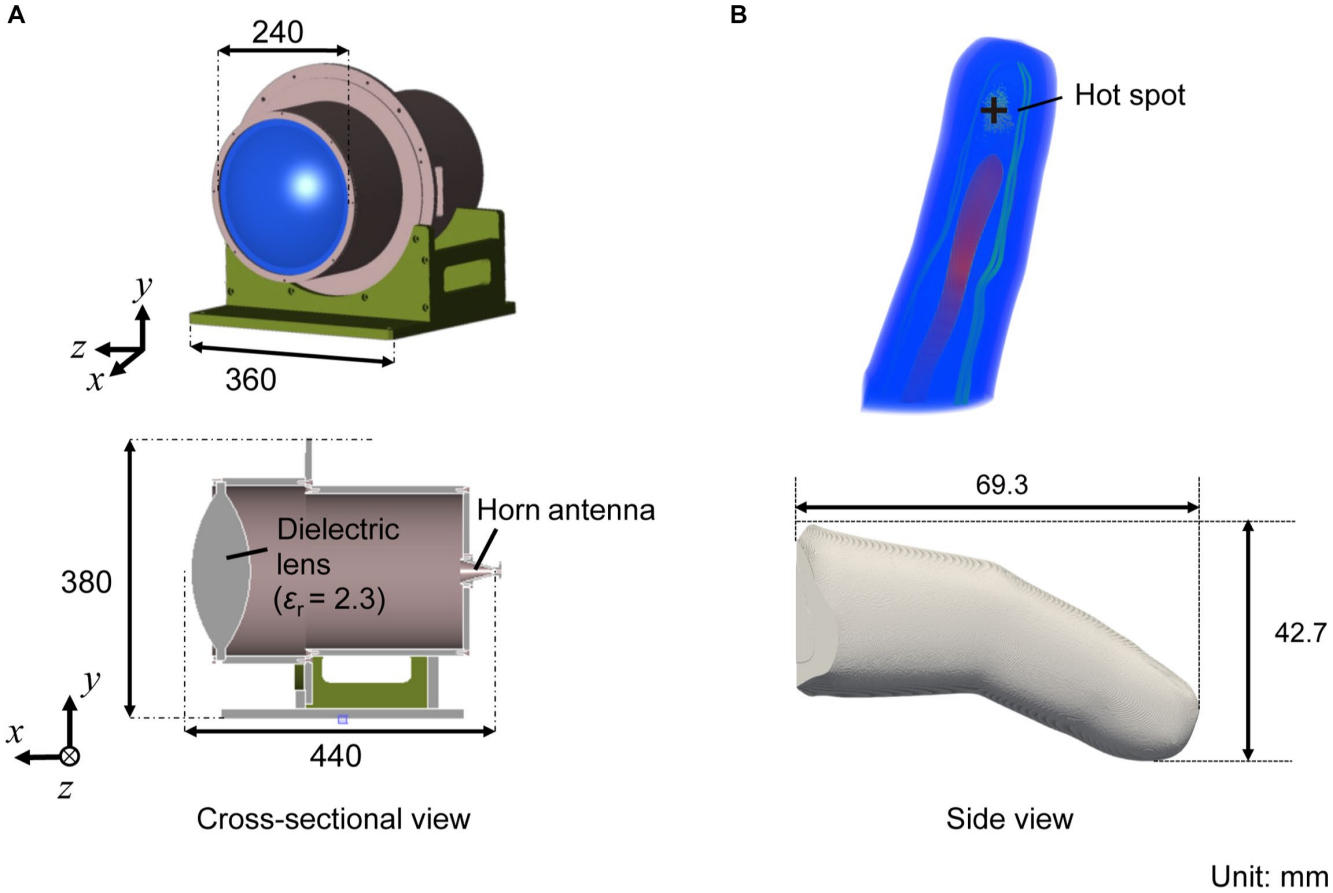
**(A)** Computational model of the lens antenna with a 2.3 of relative permittivity, and **(B)** an anatomical human hand model.

The spatial propagation of the electromagnetic field radiating from the lens antenna and the power absorbed in the fingertip were computed using a commercial software (XFdtd, Remcom, TX, United States). The computational model of the lens antenna and an anatomical hand model are depicted in Figure 3. The lens antenna consists of a horn antenna and a dielectric lens made of an ultrahigh-molecular-weight dielectric (relative permittivity of 2.3). The anatomical hand model (Murakawa et al., 2020) developed from a XCAT phantom model (50%ile value age at approximately 42 years) (Segars et al., 2010) was used to evaluate the power absorption in the fingertip. The hand model consisted of seven tissues (skin, fat, muscle, tendon, cortical bone, cancellous bone, and blood) with a resolution of 0.1 mm. The dielectric parameters were derived using the 4-Cole-Cole dispersion model (Gabriel et al., 1996) (Table 1). This single (standardized) hand model was used to compare with measurement for different participants. One reason for this simplicity is that the variability of the hand is not well characterized by morphological parameters, such as body height and weight.

**Table 1:** Dielectric properties of tissues at 28 GHz.

	Conductivity (S/m)	Relative permittivity	Density (kg/m^2^)
Skin	21.00	16.53	1,109
Fat	1.96	3.64	911
Bone cortical	4.96	5.15	1,908
Bone narrow	8.92	7.48	1,920
Blood	41.29	23.38	1,050
Tendon	28.64	19.22	1,038

### 2.4 Experimental protocol

The experiments were conducted in a quiet room with average temperature of 24.1 (SD, 1.1°C) and a relative humidity of 65.7 (SD, 9.7%). The participants were seated on a chair with their left forearm on an inflatable cushion in a neutral position while wearing headphones to minimize the perceived surrounding noise. They placed their left middle finger on a sponge to maintain the fingertip at a distance of 30 cm from the center of the lens antenna. A partition was in place to prevent the participants from viewing the examiner, as they had to guess when the stimulation was delivered. The participants were asked to maintain their sitting posture as much as possible throughout the recordings.

Prior to the experimental session, we started with a familiarization session where the participants experienced typical sensations when exposed to the millimeter waves. During the familiarization session, the left index fingertip was exposed to the millimeter waves using an input power of 1.6 W. If thermal sensation was not perceived, the session was repeated using a higher input power of 2.1 W. Consequently, all participants perceived the thermal sensation at the input power of 2.1 W, at maximum, in this session.

In the experimental session, millimeter waves were delivered to the participants’ left middle fingertips. During each trial, the skin surface temperature was recorded 1 min before the exposure for the baseline measurement. The verbal instructions were delivered 10–15 s before starting the exposure, which asked the participants to prepare for the exposure. Thereon, as soon as they perceived a certain sensation on their fingertip, the participants responded by pressing a computer mouse with their right hand. The millimeter-wave exposure was stopped when the participants clicked on the computer mouse. If the participants did not feel any sensation for 3 min at maximum, the exposure was stopped by the experimenter. Following the exposure, the skin temperature of the fingertip was checked every minute for 3 min at maximum, and the recording was stopped when the temperature declined to the baseline level (i.e., ± 1°C of the temperature at the start of the exposure). The next trial was then started.

To investigate the differences in the effects of the exposure levels, the range of the antenna input power was as wide-ranging as a very low level that would not generate sufficient heat to a high level that would produce a clear sensation: 0.2, 1.1, 1.6, 2.1, and 3.4 W. The corresponding IPD was derived from the electromagnetic computation (See Section 3.1). A single experimental session consisted of five trials with these five different exposure levels of input power were conducted in a randomized order. The experimental session was repeated twice with a short break (< 60 s) on the same day to evaluate the reliability of the perception threshold. For each participant, a total of 10 trials were conducted (five conditions × two sessions).

### 2.5 Data analysis

Since thermoreceptors are known to be activated in response to temperature changes from the baseline skin temperature (Zhang, 2015), the thermal sensation threshold was defined as the degree of change in the skin temperature from the baseline to the point at which a participant perceived a certain sensation. The baseline temperature was determined as an average of 10 s prior to the start of the exposure. If a participant perceived a thermal sensation in both of the two sessions, the mean degree of temperature change was calculated and determined as the threshold, and if a participant perceived the thermal sensation once in the two sessions, the threshold was determined from this session. The mean and standard deviation of the thresholds across participants were then calculated in each exposure condition. To clarify the difference in the degree of temperature change, we calculated the rate of temperature rise during the first 4 s of the exposure for each condition, except for the trials in which the participants did not perceive any sensation. We also measured the reaction time from the start of the exposure to when a participant perceived a sensation for each condition. The representative values for each participant for the rate of temperature rise and reaction time were also calculated in the same manner as the sensation thresholds, depending on whether a participant perceived in both sessions or in at least one session. The mean and standard deviation of those representative values across participants were calculated in each exposure condition. As an indirect index for the reliability of perception, we additionally calculated the rate of perception. We counted the number of mouse clicks (the response when participants perceived a sensation) in two sessions (i.e., 0, 1, and 2 times) for each participant under each input power condition. The number of participants who responded 0, 1, or 2 times in each condition was then counted, and the perception rate was defined as how many participants out of all perceived each number of perceptions (0, 1, and 2).

The reliability of the thermal sensation threshold between two experimental sessions was assessed for each condition. The intraclass correlation coefficient (ICC) estimates and their 95% confidence intervals were calculated to estimate the test–retest reliability. The ICC values were interpreted using the Landis and Koch interpretation of agreement (≥ 0.81 = “almost perfect,” 0.61–0.80 = “substantial,” 0.41–0.60 = “moderate,” 0.21–0.40 = “fair,” 0.00–0.20 = “slight,” and < 0.00 = “poor”) (Landis and Koch, 1977). Bland–Altman analysis was also used to confirm the presence of systematic bias, and the limits of agreement were plotted. The presence of fixed bias was confirmed when the 95% of confidence intervals of the differences between the two sessions did not include zero. The proportional bias was tested using a liner regression analysis and confirmed when the regression coefficient was not equal to zero (*p* < 0.05). All statistical analyses were conducted using SPSS statistical package version 26 (IBM Corp., Armonk, NY, United States).

## 3 Results

### 3.1 Dosimetric evaluation of electromagnetic fields radiated from antenna

**Figure 4:**
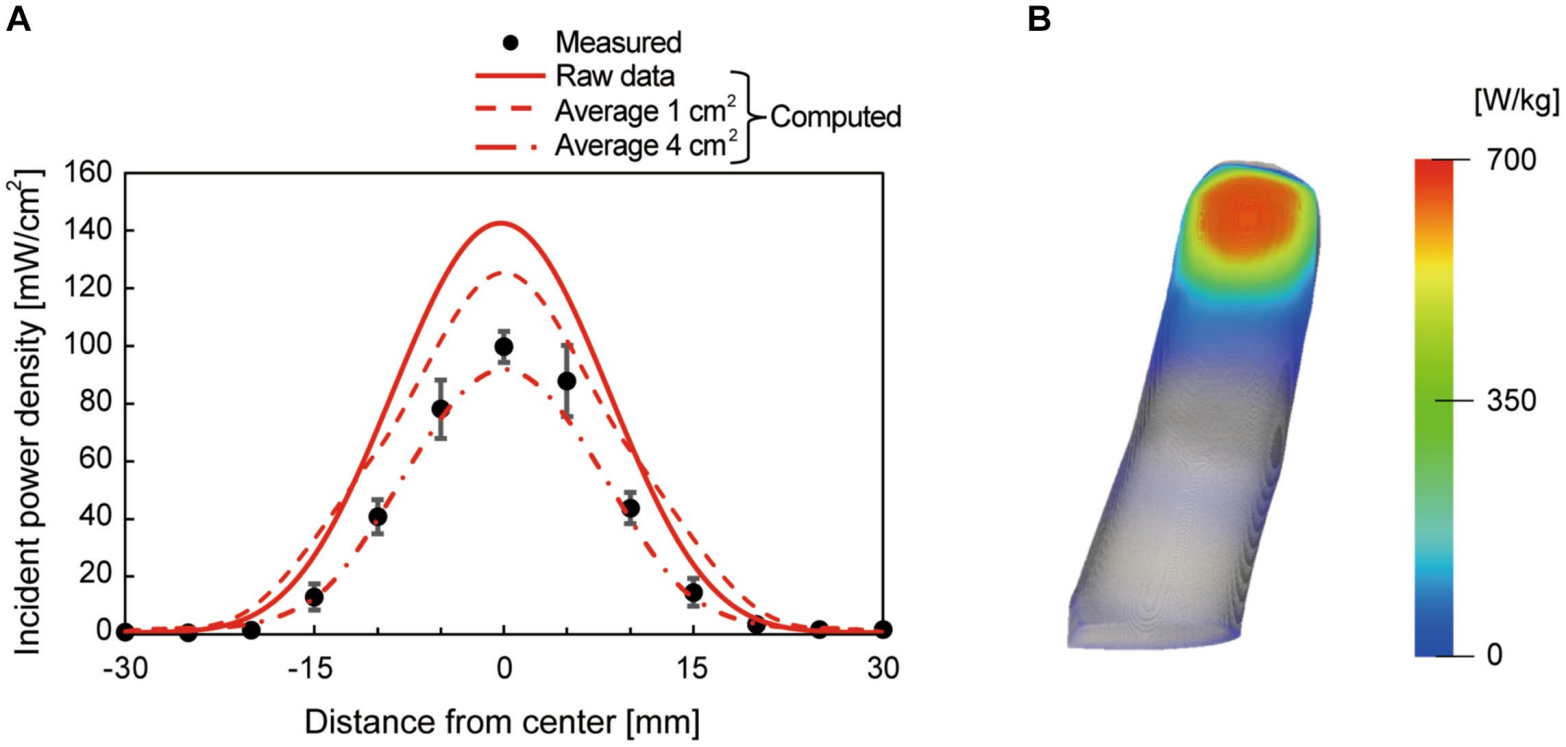
**(A)** Measured and computed incident power density at the distance of 30 cm from the antenna surface at the antenna input power of 1.1 W. The computed spatial average values (average 1 cm^2^ and average 4 cm^2^) were also plotted as references, in addition to the raw values with a resolution of 0.1 mm. The center of the lens antenna was set to 0 on the *x*-axis, and **(B)** the distribution of absorbed power (specific absorption rate) as a heatmap on a participant’s fingertip. The peak incident power density averaged over 1 cm^2^ was 126 mW/cm^2^.

Figure 4A shows the comparison between the measured and computed IPD at a distance of 30 cm from the antenna. The −3 dB bandwidth of the spatial power density was 2 cm. The measured IPD was matched with the computed values averaged over 4 cm^2^ (2 cm × 2 cm) due to the uncertainty of the measured area of the field strength meter (the physical sensor size of 6 cm in diameter). The computed peak IPD averaged over a 1 cm^2^ (1 cm × 1 cm) area was 27, 126, 191, 252, and 399 mW/cm^2^, at the antenna input power of 0.2, 1.1, 1.6, 2.1, and 3.4 W, respectively. These IPDs correspond to 0.9, 4, 6, 8, and 13 times the reference levels for occupational exposure (30 mW/m^2^ of IPD averaged over 1 cm^2^ at 30 GHz), respectively [The Institute of Electrical and Electronics Engineers (IEEE), 2019a; International Commission on Non-Ionizing Radiation Protection (ICNIRP), 2020]. The computed absorbed power density averaged over a 1 cm^2^, which is the internal physical quantity defined as the basic restriction [The Institute of Electrical and Electronics Engineers (IEEE), 2019a; International Commission on Non-Ionizing Radiation Protection (ICNIRP), 2020], was 11, 54, 82, 108, and 172 mW/cm^2^, respectively. Figure 4B shows the distribution of the power absorption in the fingertip. The beam exposure of millimeter waves produced a hotspot of a circular shape on the skin surface.

### 3.2 Thermal sensation threshold

**Figure 5:**
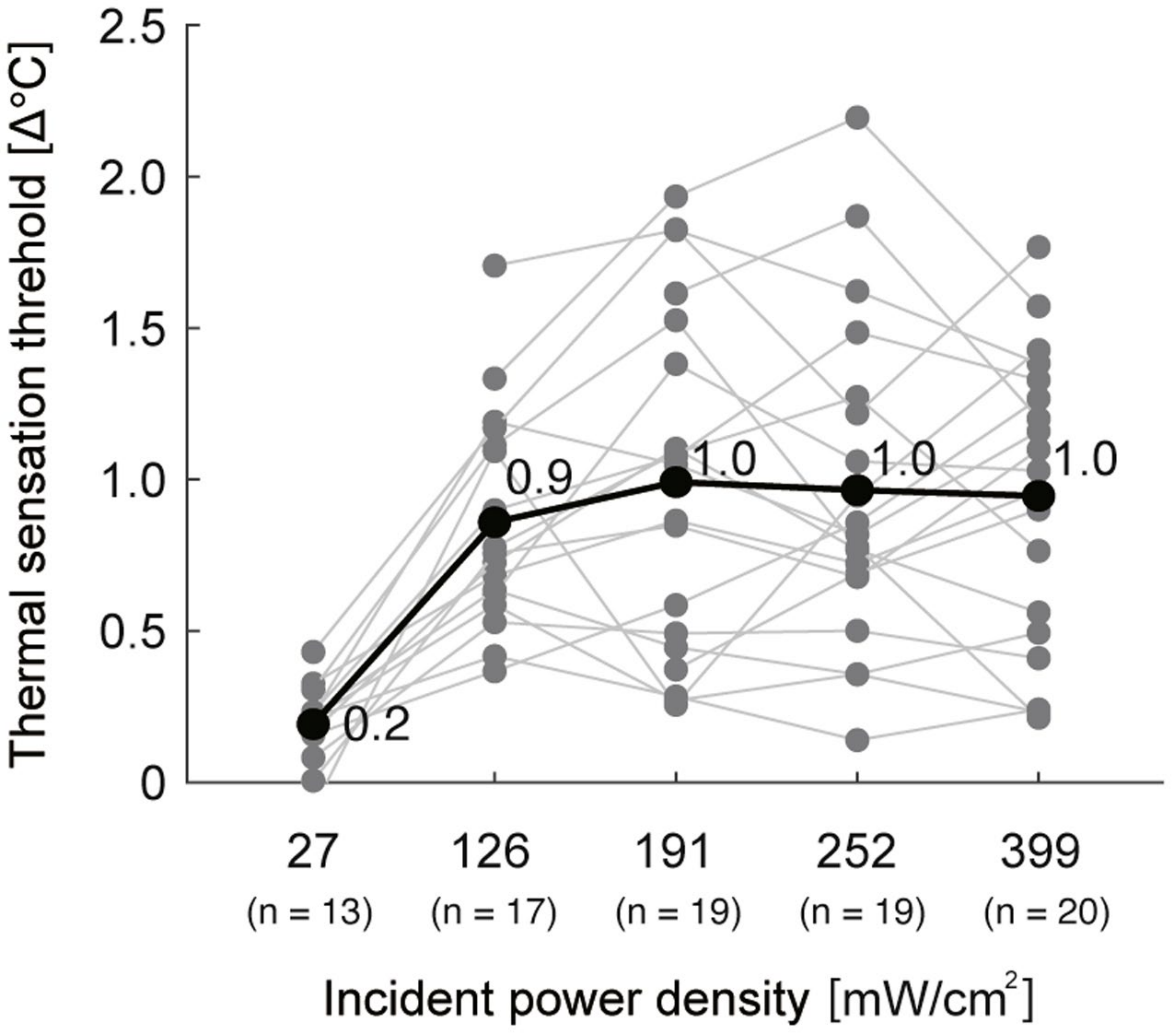
The thermal sensation thresholds for all participants. The black thick line with values represents the mean of all participants. The gray lines represent individual data. *n*: number of participants whose threshold was determined in both sessions or in at least one session in each exposure condition.

The mean baseline temperatures were 34.9 (SD, 1.0), 34.6 (1.0), 34.8 (0.9), 34.7 (1.3), and 34.8 (1.0)°C, respectively, for IPD of 27, 126, 191, 252, and 399 mW/cm^2^. The mean of the thermal sensation thresholds for each exposure condition were within 0.9–1.0°C for the exposure conditions at 126–399 mW/cm^2^ and were nearly consistent, except for 0.2°C for the 27 mW/cm^2^ condition (Figure 5; Table 2). The rate of temperature rise increased as the exposure level increased; the higher the exposure level, the faster the temperature increase (Table 3). Furthermore, the reaction time was shorter as the exposure level increased (Table 3).

**Table 2:** Mean (standard deviation) of the thermal sensation thresholds across participants.

Incident power density (mW/cm^2^)	27	126	191	252	399
Temperature change (°C)	0.19 (0.14)	0.86 (0.36)	0.99 (0.57)	0.96 (0.54)	0.95 (0.46)

If a participant perceived a thermal sensation in both of the two sessions, the mean degree of temperature change was obtained as the threshold, and if a participant perceived the thermal sensation once in the two sessions, the threshold was determined from this session.

**Table 3:** Mean (standard deviation) of the rate of temperature rise and reaction time across participants.

Incident power density (mW/cm^2^)	27	126	191	252	399
Rate of temperature rise (°C/s)	< 0.01 (0.02)	0.05 (0.01)	0.08 (0.02)	0.11 (0.03)	0.17 (0.03)
Reaction time (s)	82.6 (44.2)	45.4 (32.0)	28.8 (25.8)	13.5 (11.6)	9.7 (11.6)

The representative values of each participant were obtained in the same manner as the sensation thresholds, depending on whether a participant perceived in both sessions or in at least one session.

### 3.3 Reliability of measurements

**Figure 6:**
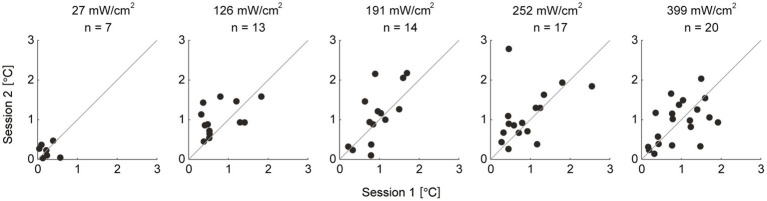
Scatterplots of the thermal sensation thresholds between two sessions for each exposure level. Each dot represents thermal sensation thresholds of each individual. The thermal sensation thresholds during session 1 are on the *x*-axis and during session 2 are on the *y*-axis. The gray lines represent *y* = *x*. *n*: number of participants whose threshold was determined in both sessions in each exposure condition.

**Figure 7:**
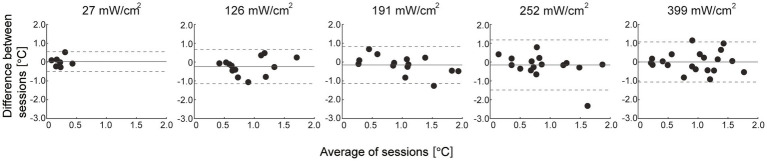
Bland–Altman plots for the thermal sensation threshold. The gray solid lines represent the mean of the difference between sessions and the dashed lines represent the upper and lower 95% limits of agreement. Each dot represents individual data.

The ICCs between sessions were “poor” at IPD of 27 mW/cm^2^ and “fair” at 126 mW/cm^2^ conditions (ICC = −0.09 and 0.39 for 27 and 126 mW/cm^2^, respectively), and “moderate” to “substantial” for the higher IPD (ICC = 0.62, 0.48, and 0.51 for 191, 252, and 399 mW/cm^2^, respectively) (Figure 6; Table 4). Bland–Altman plots showed that there was no fixed bias but a proportional bias only in the 191 mW/cm^2^ condition (*p* = 0.03) (Figure 7; Table 5). Regarding the rate of perception, all participants perceived a thermal sensation in the 399 mW/cm^2^ condition in two sessions, and the rate gradually decreased as the exposure level decreased from 252 to 27 mW/cm^2^ (Table 6). Importantly, seven and six out of 20 participants (approximately two-thirds in total) answered that, in the exposure condition at 27 mW/cm^2^, they felt thermal sensation in both or in one of the sessions, respectively. Only for trials where participants did not perceive any sensation, we retrospectively examined the maximum skin temperature change during the 3-min exposure to determine whether or not the skin temperature exceeded the sensation threshold of 1.0°C. In the trials where participants felt no sensation, the skin temperature increase reached more than 1.0°C in the conditions at IPD of 126–252 mW/cm^2^ while 0.6°C on average in the exposure at 27 mW/cm^2^ (Table 7).

**Table 4:** Mean (standard deviation) of the thermal sensation thresholds, and intraclass correlation coefficient values for reliability of the thermal sensation threshold in each condition.

Incident power density (mW/cm^2^)	Mean (SD) (°C)		ICC (1, 1)	95% CI
	1st	2nd		
27	0.24 (0.15)	0.16 (0.19)	−0.09	−0.72–0.65
126	0.79 (0.45)	0.97 (0.42)	0.39	−0.16–0.76
191	1.04 (0.48)	0.97 (0.69)	0.62	−0.18–0.86
252	0.88 (0.61)	1.03 (0.67)	0.48	−0.02–0.77
399	0.95 (0.54)	0.94 (0.54)	0.51	−0.11–0.77

CI, confidence interval; ICC, intraclass correlation coefficient; SD, standard deviation.

**Table 5:** Results for absolute reliability of the thermal sensation threshold.

Incident power density (mW/cm^2^)	27	126	191	252	399
Fixed bias					
The mean of the difference	−0.12	−0.24	−0.15	−0.15	0.01
The 95% CI of the difference	−0.26–0.19	−0.52–0.04	−0.44–0.14	−0.50–0.20	−0.25–0.26
Proportional bias					
Linear regression *B*	−0.17	0.30	−0.54	−0.20	< 0.01
*p* value	0.26	0.42	0.03^*^	0.51	1.00

CI, Confidence interval.^*^p < 0.05.

**Table 6:** The rate of perception.

Number of responses	Incident power density (mW/cm^2^)				
	27	126	191	252	399
0	7	3	1	1	0
1	6	4	5	2	0
2	7	13	14	17	20
Total	20	20	20	20	20

The number of participants who perceived a sensation in two sessions.

**Table 7:** Mean (standard deviation) of the maximum temperature rise in the trials where the participants did not perceive the thermal sensation.

Incident power density (mW/cm^2^)	27	126	191	252	399
Temperature change (°C)	0.60 (0.39)	1.34 (0.55)	1.93 (0.89)	1.72 (0.44)	–

## 4 Discussion

This study investigated the thermal sensation threshold during millimeter-wave exposure and the test–retest reliability of the measurement. Our results showed that the thermal sensation thresholds were consistent across exposure levels (approximately 1.0°C in relative temperature change), except for the lowest IPD of 27 mW/cm^2^. The relative reliability at 27 and 126 mW/cm^2^ was poor and fair, respectively, whereas that with higher electrical power was moderate to substantial. Regarding absolute reliability, a proportional bias was found only in the 191 mW/cm^2^ condition. Furthermore, the rate of perception was higher as the exposure levels increased. These results suggest that the thermal sensation threshold is nearly consistent, and its reliability depends on the level of electrical power exposure.

This study showed that there were only marginal differences in thermal sensation threshold between exposure levels at 126–399 mW/cm^2^ in relative temperature rise (0.9–1.0°C). Our results indicate that whatever the level of the exposed electrical power, thermal sensation would be perceived when the skin temperature of the exposed body part increases by more than a certain degree (approximately 1°C). In other words, the thermal sensation threshold depends on the relative temperature increase, regardless of the level of electrical power exposure. However, it cannot be ruled out that the thermal sensation threshold might also depend on the absolute temperature. As the baseline temperature was similar across conditions with different exposure levels in this study, further tests with different baseline temperatures are needed to clarify this possibility.

Previous studies have also investigated thermal sensation thresholds using different heat sources. For example, several studies applying the Peltier-based thermal stimulator demonstrated the warm sensation threshold approximately at 1.9–3.2°C in the hand (Defrin et al., 2006), 0.3–3.0°C in the forearm (Kenshalo et al., 1968), and 1.4°C in the face (Davies et al., 1983). Furthermore, a study using pulsed magnetic fields showed that the warm sensation threshold was 2.9°C in the hand (Shupak et al., 2004). The inconsistency in thermal sensation thresholds across studies, including the present, may be due to significant methodological differences in the heat source and the targeted body part.

Our results showed that the relative reliability at 27 and 126 mW/cm^2^ was poor and fair, respectively, whereas that with higher exposure levels was moderate and substantial. Additionally, the rate of perception increased with increasing exposure levels. These results suggest that the reliability of the thermal sensation threshold depends on the level of electrical power exposure.

The exposure level of the electrical power is related to the average IPD, which is defined as the quantity of power per unit area that impinges on the body surface [The Institute of Electrical and Electronics Engineers (IEEE), 2019a]. Therefore, the observed reliability of the thermal sensation threshold can be interpreted as being associated with the amount of electrical power absorbed by the exposed skin. When the human body is exposed to electrical magnetic fields, the absorbed electrical power is transmitted to the tissue, and energy is converted to heat, and the temperature of the exposed skin increases (Hashimoto et al., 2017). Thus, the greater the electrical power absorbed by the exposed skin surface, the more energy converted to heat, which activates the thermoreceptors. Consequently, this process can result in a clear and reliable perception of the sensation. Further, an increase in the rate of temperature rise with exposure levels may contribute to an increase in the rate of perception. Previous studies have shown increases in the magnitude of perception (i.e., a stronger sensation) with increasing rates of temperature rise (Davies et al., 1983; Green and Akirav, 2010). It has been suggested that more rapid heating leads to stronger afferent inputs in thermal pathways, which may cause stronger sensations (Green and Akirav, 2010). Presumably, the rapid transfer of the energy to heat could simultaneously activate a large portion of the thermoreceptors with a high discharge rate, which would produce a clear sensation of warmth (Konietzny and Hensel, 1977; Davies et al., 1983). In addition, Kenshalo et al. (1968) reported a decrease in human temperature sensitivity when the rate of stimulus temperature was slower than rates of 0.1°C/s, likely due to thermal adaptation. Given that the rates of temperature rise during the exposure at 27–126 mW/cm^2^ conditions were 0.05°C/s or slower in this study, low reliability of the perception threshold in the conditions with lower electrical power may be associated with thermal adaptation during the exposure which could result in a less distinct perception of the thermal sensation. Our results also showed that the ICC of the thermal sensation threshold at 191 mW/cm^2^ was the highest among the conditions, but a proportional bias was found between the sessions upon plotting the Bland–Altman graph. The plot shows that the thermal sensation thresholds during the second session tend to be higher than that during the first session, which may be attributed to the thermal adaptation in the second session. Although the reason why only the exposure at 191 mW/cm^2^ resulted in this trend is unclear, it is assumed that the sensation perceived during this condition may be moderate between clear and vague, which may result in thermal adaptation. In fact, the increase in skin temperature reached nearly 2.0°C during the non-response trials in the exposure at 191 mW/cm^2^. This result also supports that the moderate temperature rise (0.08°C/s) in this condition may be associated with inducing thermal adaptation through the sessions.

It can be argued that the low ICC at 27 mW/cm^2^ condition is due to the low variability of the thresholds between participants, and thus the ICC result cannot simply be interpreted as that the exposure condition at 27 mW/cm^2^ is unreliable. However, our results showed that one-third of participants responded that they felt the thermal sensation in both sessions, one-third felt it once, and the remaining one-third felt nothing twice in the exposure at 27 mW/cm^2^. We believe that these variable responses illustrate the low reliability of this condition rather than simply the low variability of the data. Besides, it should be noted that approximately two-thirds of participants responded that they felt the sensation at least once, despite the lowest IPD of 27 mW/cm^2^. Although it is unknown whether the participants were sensitive to the millimeter waves and actually perceived a sensation, the findings that the rate of temperature rise was quite slow (< 0.01°C/s) and they responded at a temperature increase of only 0.2°C on average, much less than that in the other conditions, suggest that their responses might be attributed to a bias in the expectation of perception. In fact, the maximum temperature increase during the non-response trials was less than 1.0°C, suggesting that the exposure level at IPD of 27 mW/cm^2^ was insufficient to produce thermal sensation. In addition, we can interpret these results as indicating that the perception of thermal sensation is inherently ambiguous owing to the nature of human perception. Our perception is a subjective experience of interpreting a sensation, which is influenced by one’s own knowledge, past experiences, and thoughts related to stimuli (Dumper et al., 2019). Therefore, it should be noted that the psychological and physical states of a person can easily affect the evaluation of the perception of thermal sensation. This nature of perception may attribute to “moderate” relative reliability in the exposure conditions at 252 and 399 mW/cm^2^ despite the higher exposure levels in the present conditions.

This study has some limitations. First, the difference in the ICC between exposure levels was not statistically tested. Although using the mixed effects model enabled us to test this, the modeling was not applied because the limited sample size would lead to low estimation accuracy. This point should be addressed in future studies with larger sample sizes. Secondly, this study recruited participants from a limited age range of young to middle-aged adults. Therefore, it is uncertain whether our findings are generalizable to other aging populations. We speculate that the rate of perception of thermal sensation would decrease in the elderly owing to age-related peripheral morphological changes and/or central factors such as cognitive functions (Uehara et al., 2023).

## 5 Conclusion

The thermal sensation thresholds induced by the exposure to millimeter waves are constant at the temperature of approximately 1.0°C at different exposure levels over a certain input power (IPD of 27 mW/cm^2^ in this study), whereas its reliability increases with higher exposure levels (IPD of 252 mW/cm^2^ or higher in this study). Exposure to higher levels of electrical power may be appropriate while studying the characteristics of perceptions and their thresholds induced by the exposure to millimeter waves in future investigations.

## Data availability statement

The original contributions presented in the study are included in the article, further inquiries can be directed to the corresponding authors.

## Ethics statement

The studies involving humans were approved by the Ethics Review Committee, Fujita Health University. The participants provided their written informed consent to participate in this study.

## Author contributions

AY: Investigation, Data curation, Formal analysis, Methodology, Visualization, Writing – original draft, Writing – review & editing. SU: Conceptualization, Methodology, Project administration, Writing – original draft, Writing – review & editing. KU: Investigation, Data curation, Methodology, Writing – original draft. SK: Conceptualization, Formal analysis, Resources, Visualization, Writing – review & editing, Software. NA: Formal analysis, Visualization, Writing – review & editing, Software. AH: Conceptualization, Funding acquisition, Project administration, Resources, Supervision, Writing – review & editing. YO: Conceptualization, Funding acquisition, Project administration, Resources, Supervision, Writing – review & editing.
